# Global heart failure incidence rate: an updated systematic review and meta-analysis

**DOI:** 10.3389/fcvm.2026.1714070

**Published:** 2026-03-20

**Authors:** Jhosmer Ballena-Caicedo, Fiorella E. Zuzunaga-Montoya, Lupita Ana Maria Valladolid-Sandoval, Julio César Bautista Zuta, Mario J. Valladares-Garrido, Carmen Inés Gutierrez De Carrillo, Darwin A. León-Figueroa, Víctor Juan Vera-Ponce

**Affiliations:** 1 Instituto de Investigación de Enfermedades Tropicales, Universidad Nacional Toribio Rodríguez de Mendoza de Amazonas (UNTRM), Amazonas, Perú; 2 Facultad de Medicina (FAMED), Universidad Nacional Toribio Rodríguez de Mendoza de, Amazonas (UNTRM), Amazonas, Perú; 3 Universidad Continental, Lima, Perú; 4 Hospital Regional Virgen de Fátima, Chachapoyas, Perú; 5 Escuela de Medicina Humana, Universidad Señor de Sipán, Chiclayo, Peru; 6 Facultad de Medicina Humana, Universidad de San Martín de Porres, Chiclayo, Peru; 7 EpiHealth Research Center for Epidemiology and Public Health, Lima, Peru

**Keywords:** epidemiology, global health, heart failure, incidence, meta-analysis, systematic review

## Abstract

**Introduction:**

Heart failure (HF) affects 64 million people worldwide, yet updated global estimates of its incidence are lacking.

**Objective:**

Conduct a systematic review (SR) and meta-analysis to estimate the global incidence of heart failure and analyze its variations according to geographic region, sex, age, and clinical characteristics.

**Methodology:**

A SR was performed in MEDLINE (PubMed), Scopus, EMBASE, and Web of Science (including SciELO) for observational studies published between 2000 and 2025 reporting HF incidence rates in the general population. Studies with recognized diagnostic criteria (Framingham, ESC, ACC/AHA) or standardized codifications (ICD) were included. The meta-analysis employed a random-effects model with the REML method. Subgroup analyses (sex, age, region, phenotypes) and meta-regression were conducted to explore temporal trends.

**Results:**

Forty-two studies (2001–2025) from 18 countries were included, with 57% categorized as low risk of bias. The combined global incidence was 2.72 cases per 1,000 person-years (95% CI: 1.95–3.81), being higher in North America (6.06) than in Europe (2.65) and Asia (4.08). Incidence was higher in men (1.93) than in women (1.62), and substantially higher in the population >50 years (12.38). Meta-regression by publication year showed no significant temporal trend (*p* = 0.255); however, when the midpoint of the data collection period was used, a significant declining trend was identified (*p* = 0.009, *R*^2^ = 23.75%).

**Conclusions:**

This meta-analysis provides updated global HF incidence estimates, revealing significant regional disparities and rates quadrupling in the population over 50 years. However, the pooled estimate is predominantly derived from high-income countries, limiting generalizability to low- and middle-income settings.

## 1 Introduction

Heart failure (HF) constitutes a complex clinical syndrome characterized by symptoms (dyspnea, fatigue, peripheral edema) and typical signs (jugular engorgement, pulmonary crackles, edema) derived from structural or functional cardiac abnormalities that cause elevated intracardiac pressures and/or inadequate cardiac output (1). This syndrome represents one of the main challenges for global healthcare systems, with an estimated prevalence of 64 million people affected worldwide (2, 3). The clinical and economic consequences are considerable: HF is associated with high mortality (approximately 50% at 5 years from diagnosis), significant deterioration in quality of life, functional limitation, and high healthcare costs, estimated at more than 346 billion dollars annually (4). In response to this impact, the 2021 European Society of Cardiology (ESC) guidelines and the 2022 American College of Cardiology/American Heart Association (ACC/AHA) update have refined the definition, classification, and therapeutic approach to this syndrome, emphasizing the need for optimal, personalized management (1, 5).

The epidemiology of HF has experienced significant changes in recent decades, with divergent patterns according to region. While age-adjusted incidence tends to stabilize and even decrease in some developed countries, global prevalence continues to rise, driven by population aging, increased survival after acute cardiovascular events, and more effective therapies that prolong life in patients with HF (6, 7). Recent analysis from the Global Burden of Disease (GBD) 2021 documented an increase in the age-standardized global prevalence from 647.9 per 100,000 in 1990 to 682.7 per 100,000 in 2021, with 56.5 million prevalent cases worldwide (8). Regional disparities are notable: the North Africa and Middle East region has the highest standardized prevalence (780.5 per 100,000), while South Asia records the lowest (600.1 per 100,000) (8). Concurrently, the clinical profile has evolved, with a progressive increase in the proportion of patients with heart failure with preserved ejection fraction (HFpEF), which now represents approximately 50% of cases, posing additional therapeutic challenges given that treatments that significantly improve prognosis are still lacking for this phenotype (9, 10).

Despite abundant research on HF, important gaps persist in the consolidation of epidemiological data at a global level. Evidence predominantly comes from industrialized nations, with limited representation from low- and middle-income regions. A recent systematic review found that only 11 of 51 countries with population-based studies on HF belonged to low- or middle-income regions, contributing just about 10% of the observed person-years (11). This unequal distribution makes it difficult to obtain a complete picture of the incidence of HF worldwide, particularly in regions where the causes and characteristics of the syndrome may differ considerably from those described in Western countries (12). Although previous systematic reviews have evaluated specific aspects of the epidemiology of HF (13, 14), to date none has provided a global and updated synthesis of HF incidence that covers all geographic regions and considers variations by age, sex, and clinical phenotypes.

Recognizing this need, the present work aims to conduct a systematic review (SR) and meta-analysis of the literature to estimate the updated global incidence of heart failure. The goal is to synthesize available evidence from different countries and regions, obtaining comparable estimates that allow more precise dimensioning of the magnitude of the problem in different contexts. Having consolidated global data has a clear clinical and public health justification: it will facilitate decision-making and healthcare resource planning based on the actual burden of HF in each setting, providing robust epidemiological inputs for the design of prevention strategies and evidence-informed health policies. Ultimately, this SR aims to contribute to filling current knowledge gaps, providing a solid database on the worldwide incidence of HF that supports improving patient prognosis and guides effective actions at both clinical and global health levels.

## 2 Methodology

### 2.1 Study design

A SR with meta-analysis was conducted following the PRISMA guidelines (15) adapted for incidence studies (16, 17) (Supplementary Material S1). Standardized procedures were applied for searching, selecting, extracting, and analyzing epidemiological data on HF incidence in the general population.

### 2.2 Search strategy

The bibliographic search was conducted in MEDLINE (via PubMed), Scopus, EMBASE, and Web of Science (including SciELO), selected for their multidisciplinary and geographical coverage, in accordance with Cochrane manual recommendations (18). These databases allow retrieval of population-based studies from different regions of the world.

MeSH terms and keywords were used, combined with Boolean operators: “Heart Failure”, “Congestive Heart Failure”, “Incidence”, “Epidemiology”, connected with “OR” and “AND”. Articles published between January 2000 and April 2025 were searched, with no language restrictions. The complete search strategy is presented in Supplementary Material S2.

### 2.3 Selection criteria

Observational studies of cohort type, both prospective and retrospective, reporting HF incidence rates in the general adult population (≥18 years) were included. Studies using recognized clinical criteria (Framingham, ESC, ACC/AHA) or standardized diagnostic codes (ICD-9, ICD-10, Read codes) were admitted (1, 5). Studies had to explicitly report the number of incident cases and the corresponding denominator. Publications in any language within the period 2000–2025 were accepted.

Studies focused exclusively on specific clinical populations (e.g., hospitalized patients or those with chronic diseases), systematic reviews, meta-analyses, case-control studies, case reports, letters to the editor, and studies without a clearly specified diagnostic criterion were excluded. Articles whose main objective was not to estimate the incidence of HF were also excluded.

### 2.4 Study selection process

Search results were imported into Rayyan software for reference management and duplicate removal. Two reviewers independently evaluated titles and abstracts in blind mode. Subsequently, the full texts of potentially eligible studies were reviewed. Discrepancies were resolved by consensus or, if necessary, with the intervention of a third reviewer.

The entire selection process was documented, and a PRISMA diagram was elaborated detailing the included and excluded studies, along with the reasons for exclusion at the full-text stage. A systematic record of each phase of the process was maintained.

### 2.5 Data extraction

Data were extracted by two researchers independently using a standardized template in Microsoft Excel 2023. Information was collected on authors, year, country, study design, collection period, sample size and characteristics, sampling method, diagnostic criteria used, and main results related to HF incidence.

Discrepancies between reviewers were resolved by cross-review. In studies that did not report confidence intervals for incidence rates, these were calculated when possible, using standard formulas.

### 2.6 Risk of bias assessment

Risk of bias assessment was performed using the Muun et al. tool (16, 17), designed for observational studies of prevalence and incidence. This tool considers eight key domains: representativeness, sampling quality, diagnostic validity, exclusion of prevalent cases, data completeness, accuracy of numerator and denominator, estimation method, and source transparency.

Each domain was rated with a binary score (0 or 1), with a total score of 0 to 8. Studies were classified as low risk (7–8 points), moderate risk (4–6 points), or high risk (<4 points). Two reviewers performed the assessments independently, and differences were resolved by consensus. These assessments were considered in sensitivity analyses and qualitative interpretation of the results.

### 2.7 Statistical analysis

We used the incidence rates per 1,000 person-years as reported in each primary study rather than recalculating person-years from summary measures of follow-up time (mean, median, or maximum). This decision was based on the fact that reported rates preserve the original person-time denominators derived from individual-level data within each study. Recalculating person-years by multiplying the sample size by a summary measure of follow-up would assume uniform follow-up across all participants and would not accurately reflect individual-level censoring, loss to follow-up, or staggered entry, which are already accounted for in the reported rates. This strategy is consistent with established recommendations for meta-analysis of rates when direct access to individual participant data is not available (19, 20).

Incidence rates were log-transformed to stabilize the variance and satisfy the normality assumptions of the random-effects model, as recommended for meta-analysis of count-based outcomes (21). The sampling variance of each log-transformed rate was approximated as the inverse of the number of incident cases {Var[ln(rate)] = 1/d, where d = number of events}, a standard approximation derived from the Poisson distribution for count data that has been formally described and widely applied in meta-analyses of incidence rates (19, 21, 22). We employed a random-effects model using the restricted maximum likelihood (REML) method due to the expected heterogeneity between studies. All analyses were conducted using the metafor package in R version 4.2.0 (21).

We evaluated heterogeneity between studies using the *I*^2^ statistic, considering values above 75% as indicative of considerable heterogeneity. To explore sources of heterogeneity, we performed subgroup analyses by geographic region, publication decade, population type, sex, age group, and HF phenotype (HFrEF and HFpEF), as well as by diagnostic criteria type (administrative, clinical, or mixed), data source (administrative database or clinical cohort), study design (prospective, retrospective, or cross-sectional), and risk of bias (low or moderate). Meta-regressions were conducted to evaluate the association between incidence rates and publication year, study midpoint (midpoint of the data collection period), sample size (log-transformed), follow-up duration, diagnostic criteria type, data source, study design, and type of follow-up time measure (mean, median, or unspecified). Publication bias was assessed visually through a funnel plot and formally using Egger's regression test and Begg's rank correlation test, supplemented by the trim-and-fill method.

Additionally, we conducted several sensitivity analyses to evaluate the robustness of our findings to methodological decisions. First, we compared the pooled estimates obtained using reported incidence rates with those derived from the traditional approach, which recalculates person-years as sample size multiplied by the reported follow-up time. Second, we repeated the main meta-analysis excluding studies that did not explicitly specify the type of follow-up time measure. Third, we restricted the analysis to studies classified as low risk of bias. Fourth, we compared results across five different heterogeneity variance estimators: REML, DerSimonian-Laird, REML with Hartung-Knapp adjustment, Paule-Mandel, and Sidik-Jonkman. All results are presented as rates per 1,000 person-years with 95% confidence intervals.

## 3 Results

### 3.1 Article selection diagram

**Figure 1:**
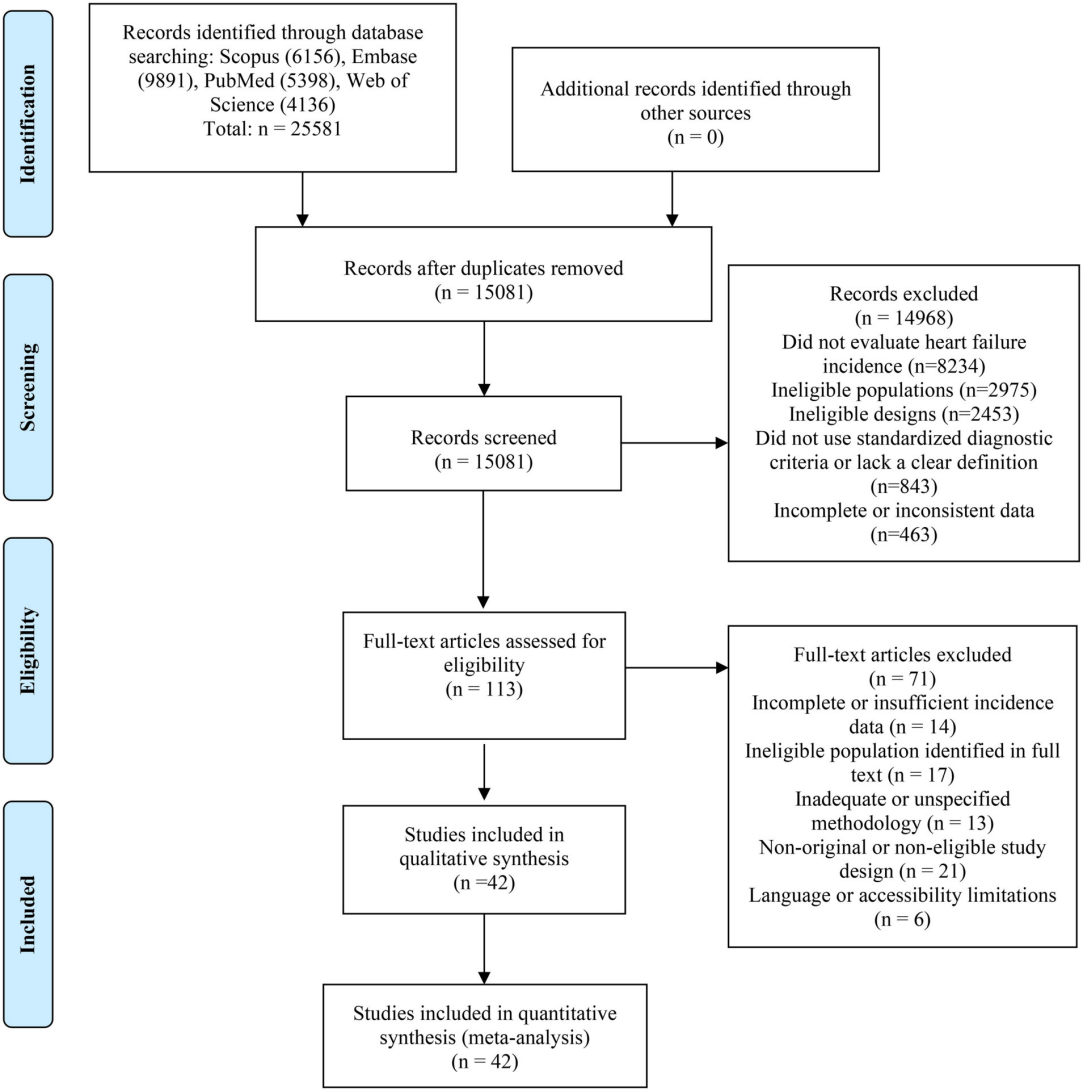
Flowchart of study selection.

A systematic search was conducted in the Scopus (*n* = 6,156), Embase (*n* = 9,891), PubMed (*n* = 5,398), and Web of Science (*n* = 4,136) databases, initially identifying 25,581 records. After removing duplicates, 15,081 articles were obtained for title and abstract review, with 14,968 excluded for not evaluating heart failure incidence (*n* = 8,234), ineligible populations (*n* = 2,975), inadequate designs (*n* = 2,453), absence of standardized diagnostic criteria (*n* = 843), or incomplete data (*n* = 463). Of the 113 articles evaluated in full text, an additional 71 were excluded mainly due to insufficient incidence data (*n* = 14), ineligible populations identified in full text (*n* = 17), inadequate methodology (*n* = 13), non-original designs (*n* = 21), or language/accessibility limitations (*n* = 6). Finally, 42 studies met the inclusion criteria and were incorporated into both the qualitative analysis and quantitative meta-analysis (23–64) (Figure 1).

### 3.2 General characteristics of included studies

The 42 included studies were published between 2001 and 2025, as presented in Supplementary Material S3. An increasing trend in scientific production on HF incidence was observed from the 2010 s onwards, with a notable concentration of publications between 2015 and 2024. 70% of the studies were published during this period.

The majority of studies corresponded to prospective or retrospective cohorts (86%), while 9% were cross-sectional studies and 5% combined cross-sectional design with longitudinal follow-up. Regarding sampling type, 56% of the studies employed probabilistic methods (26–28, 30–32, 34, 39, 43–57, 59–64), including random, multistage, or representative sampling, while 44% used non-probabilistic sampling (23–25, 29, 33, 35–38, 40–42, 58), commonly in studies based on clinical or administrative records. Sample sizes varied widely, from 393 to more than 49 million participants, with a median of 3,416 participants (interquartile range: 2,100–14,000).

The evaluated populations were predominantly adult, with ages between 18 and 100 years. 70% of the studies included middle-aged to older adults (40 to 80 years), while 20% focused exclusively on older adults (≥65 years). Only 10% analyzed cohorts with a significant fraction of young adults (25–45 years). In terms of sex, 90% of the studies evaluated mixed populations, with similar proportions of men and women; only one study focused exclusively on males. The study setting was primarily urban or metropolitan, with 18% of studies including both urban and rural settings.

Regarding diagnostic criteria, 42% of the studies used administrative coding based on international classification systems (ICD-9, ICD-10, Read codes), while 37% employed adjudicated clinical definitions, including Framingham, ESC, ACC/AHA criteria or specialized medical panels. 14% used mixed approaches, integrating ICD coding with additional clinical validation. Finally, 7% applied their own operational definitions adapted to specific registries or data derived from medical prescriptions. Diagnostic sources included hospital records, death certificates, primary care databases, clinical evaluations, and echocardiography.

### 3.3 Risk of bias assessment

Risk of bias was assessed using the Munn tool for prevalence studies across nine methodological domains (Supplementary Material S4). Of the 42 included studies, 24 (57.1%) were classified as low risk of bias (score 7–9) and 18 (42.9%) as moderate risk (score 4–6); no study was classified as high risk. The domains with the highest compliance were standard and reliable measurement of the condition (Q7, 97.6%), sufficient coverage of the identified sample (Q5, 95.2%), and appropriate statistical analysis (Q8, 92.9%). Valid methods for identification of the condition (Q6) were fulfilled in 88.1% of studies. The main methodological limitations were observed in the adequacy of response rate management (Q9, 4.8%), reflecting that most studies used administrative databases or cohorts where individual response rates were not applicable or not reported. Sample frame appropriateness (Q1, 59.5%) and appropriate sampling (Q2, 69.0%) were also relatively lower, as several studies were based on single regions, insurance databases, or volunteer cohorts that may not be fully representative of the general population. A sensitivity analysis restricted to studies with low risk of bias (*n* = 24) produced a pooled estimate of 2.69 per 1,000 person-years (95% CI: 1.67–4.31), consistent with the primary estimate.

### 3.4 Meta-analysis of global incidence and subgroup analysis

The general meta-analysis, which integrated 42 studies with data on HF incidence in the general population, yielded a combined rate of 2.72 cases per 1,000 person-years (95% CI: 1.95–3.81) (Table 1). Heterogeneity was high (*I*^2^ = 100%), indicating considerable variability between studies, probably attributable to differences in design, evaluated populations, diagnostic criteria, and data sources. When restricting the analysis to studies focused on population <50 years (*n* = 38) (23, 25, 26, 28–38, 40–55, 57–64), the rate was similar: 3.05 (95% CI: 2.41–3.86), while in the few studies that exclusively evaluated population >50 years (*n* = 4) (24, 27, 39, 56), the incidence was substantially higher: 12.38 (95% CI: 9.16–16.73).

**Table 1:** Pooled incidence of heart failure per 1000 person-years, stratified by population characteristics, clinical subtype, geographic region, and study design.

Analysis	Number of studies	Incidence per 1,000 person-years (95% CI)	*I*^2^ (%)
Overall	42	2.72 (1.95–3.81)	100.00%
General population			
Population <50 years	38	3.05 (2.41–3.86)	100.00%
Population >50 years	4	12.38 (9.16–16.73)	97.60%
Sex			
Men	28	1.93 (1.42–2.62)	100.00%
Women	28	1.62 (1.14–2.29)	100.00%
Heart failure subtype			
HFrEF	4	1.77 (0.56–5.60)	99.80%
HFpEF	4	1.58 (0.41–6.06)	99.90%
Region			
Asia	4	4.08 (1.64–10.16)	99%
Europe	26	2.65 (1.94–3.61)	98%
Global	1	5.18 (5.04–5.32)	—
North America	11	6.06 (4.38–8.37)	99%
Country			
Germany	2	4.77 (1.56–14.53)	96%
Canada	1	6.43 (6.39–6.47)	—
China	2	3.91 (0.87–17.60)	96%
South Korea	1	9.87 (9.75–9.99)	—
Scotland	1	1.99 (1.84–2.16)	—
United States	10	6.02 (4.21–8.60)	98%
Finland	1	3.20 (3.15–3.25)	—
Italy	1	1.99 (1.91–2.08)	—
Japan	1	1.84 (1.71–1.97)	—
Norway	2	7.65 (1.61–36.36)	99%
Netherlands	3	5.60 (1.23–25.44)	99%
United Kingdom	12	1.72 (1.25–2.38)	96%
Sweden	4	2.64 (2.04–3.42)	99%
Decade			
2000 s	6	4.19 (1.82–9.65)	99%
2010 s	17	2.92 (1.95–4.37)	99%
2020 s	19	3.83 (2.76–5.33)	99%
Sampling			
Non-probabilistic	13	2.67 (1.75–4.07)	96%
Probabilistic	29	3.92 (2.91–5.28)	97%

When analyzing by sex, the incidence was slightly higher in men (1.93 per 1,000 person-years; 95% CI: 1.42–2.62) (23–29, 34, 35, 37, 39, 41, 44–52, 54, 55, 58–60, 63, 64) than in women (1.62; 95% CI: 1.14–2.29) (23–30, 34, 35, 37, 39, 41, 44–52, 54, 55, 59, 60, 63, 64), although the difference was not wide. Heterogeneity was equally high in both groups (*I*^2^ = 100%), suggesting significant variability among the included studies, possibly linked to differences in age, comorbidities, and access to diagnosis. When evaluating HF subtypes, the combined incidence was 1.77 (95% CI: 0.56–5.60) for heart failure with reduced ejection fraction (HFrEF) and 1.58 (95% CI: 0.41–6.06) for preserved ejection fraction (HFpEF) (34, 39, 47, 57), both with very high heterogeneity (*I*^2^ > 99%).

The analysis by regions showed important differences. In North America (11 studies) (28, 31, 34, 39, 43, 46–48, 50, 53, 58), the rate was 6.06 per 1,000 person-years (95% CI: 4.38–8.37), clearly higher than that observed in Europe (26 studies, 2.65; 95% CI: 1.94–3.61) (23–27, 29, 30, 32, 33, 35–38, 40, 42, 44, 45, 49, 54, 55, 57, 59, 60, 62–64) or Asia (4 studies, 4.08; 95% CI: 1.64–10.16) (51, 52, 56, 61). These regional differences may be due to both real variations in disease burden and methodological factors (data sources, diagnostic definition, age of cohorts). At the country level, the United States (10 studies) (28, 31, 34, 39, 43, 47, 48, 50, 53, 58) presented a combined rate of 6.02 (95% CI: 4.21–8.60), Canada 6.43 (95% CI: 6.39–6.47) (46), South Korea 9.87 (95% CI: 9.75–9.99) (61), and the United Kingdom 1.72 (95% CI: 1.25–2.38) (23, 26, 35, 38, 44, 55, 57, 59, 60, 62–64), Japan (51), Italy (36), Finland (40), and Scotland (25) showed rates below 3, while Norway (49, 54) and the Netherlands (24, 27, 42) presented higher estimates but with wide intervals.

The evolution by decade showed a slight oscillation in incidence rates: in the 2000 s (*n* = 6), the combined rate was 4.19 (95% CI: 1.82–9.65) (23–28), in the 2010 s (*n* = 17) it was 2.92 (95% CI: 1.95–4.37) (29–45), and in the 2020 s (*n* = 19) it was 3.83 (95% CI: 2.76–5.33) (46–64). These variations do not show a clear trend of increase or decrease over time, although they could be influenced by changes in diagnostic criteria, population aging, or improvements in cardiovascular prevention. Finally, the analysis according to sampling type showed that studies with probabilistic design (*n* = 29) reported a combined incidence of 3.92 (95% CI: 2.91–5.28) (26–28, 30–32, 34, 39, 43–57, 59–64), slightly higher than that of non-probabilistic studies (*n* = 13), whose rate was 2.67 (95% CI: 1.75–4.07) (23–25, 29, 33, 35–38, 40–42, 58).

#### 3.4.1 Meta-regression analysis by publication year

**Figure 2:**
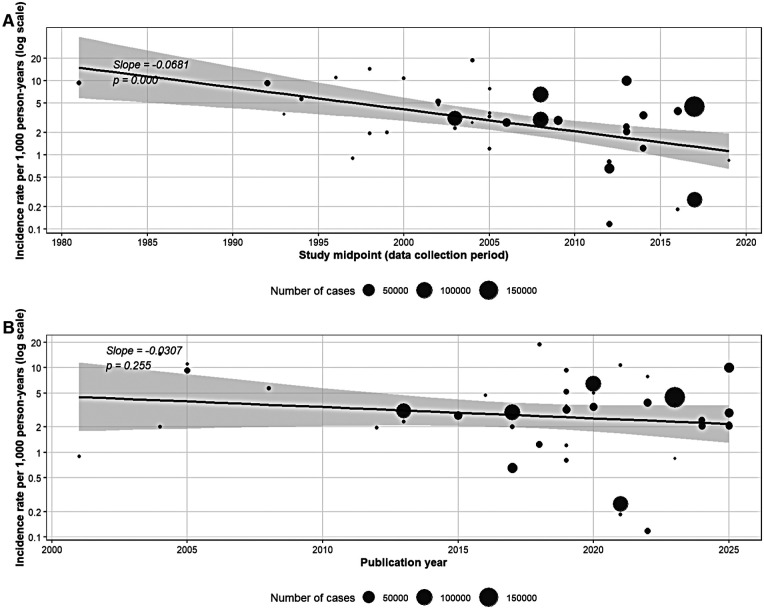
Meta-regression of HF incidence rates by study midpoint **(A)** and publication year **(B)**.

A meta-regression was performed to explore the possible relationship between the publication year of the studies and the reported HF incidence rates (Figure 2A). The slope estimated by the model was negative but did not reach statistical significance (coefficient = −0.031, *p* = 0.255, *R*^2^ = 0.72%). However, when the analysis was repeated using the midpoint of the data collection period rather than the publication year, the association was statistically significant (coefficient = −0.068, *p* = 0.009, *R*^2^ = 23.75%) (Figure 2B), suggesting a declining temporal trend in HF incidence that is better captured by the actual period of data collection than by the year of publication. This distinction is relevant because the lag between data collection and publication can span several years, and publication year may not accurately reflect the epidemiological period under study. Additional meta-regressions were performed to evaluate the influence of sample size (log-transformed), follow-up duration, diagnostic criteria type, data source, and study design on the pooled estimates. Of these, sample size (*p* = 0.006, *R*^2^ = 13.79%) and follow-up duration (*p* = 0.039, *R*^2^ = 7.35%) showed significant associations, while diagnostic criteria, data source, and study design did not significantly explain the observed heterogeneity. A complete summary of all meta-regressions is presented in Supplementary Material S5.

### 3.5 Publication bias assessment

Visual inspection of the funnel plot showed no evident asymmetry in the distribution of study estimates (Supplementary Material S6). This was confirmed by both Egger's regression test (*p* = 0.805) and Begg's rank correlation test (*p* = 0.319), indicating no significant publication bias. The trim-and-fill method did not identify missing studies, further supporting the robustness of the pooled estimate against potential publication bias.

### 3.6 Sensitivity analyses

To evaluate the robustness of the pooled estimates, we conducted several additional analyses addressing the potential influence of methodological decisions on the results. First, we compared our primary analytical approach (using reported incidence rates) with the traditional method, which recalculates person-years as sample size multiplied by follow-up time. The traditional approach (*n* = 42) produced a pooled estimate of 3.48 per 1,000 person-years (95% CI: 2.72–4.46), consistent in direction and magnitude with the primary estimate of 2.72 per 1,000 person-years (95% CI: 1.95–3.81) (Supplementary Material S7). Second, restricting the analysis to studies that explicitly reported a defined follow-up measure (mean or median, *n* = 7) yielded a pooled rate of 3.42 per 1,000 person-years (95% CI: 1.86–6.27), also consistent with the primary estimate (Supplementary Material S8). Third, a meta-regression assessing whether the type of summary measure for follow-up duration (mean, median, maximum, or unspecified) influenced the pooled incidence estimates showed no significant effect (QM = 0.65, df = 3, *p* = 0.885; *R*^2^ = 0.00%). Fourth, restricting the analysis to studies with low risk of bias (*n* = 26) produced a pooled estimate of 2.69 per 1,000 person-years (95% CI: 1.67–4.31), nearly identical to the primary estimate. Fifth, comparison across five different heterogeneity variance estimators (REML, DerSimonian-Laird, REML with Hartung-Knapp adjustment, Paule-Mandel, and Sidik-Jonkman) yielded identical pooled rates of 2.72 per 1,000 person-years with overlapping confidence intervals (Supplementary Material S9). These convergent results confirm that the analytical decisions did not systematically bias the findings.

## 4 Discussion

### 4.1 Main findings

This meta-analysis gathered information from 42 studies on the incidence of heart failure in the general adult population, showing a combined rate of 2.72 cases per 1,000 person-years (95% CI: 1.95–3.81). Among the main findings, the variation by age stands out, with higher rates in studies focused on older adults, as well as differences by sex and clinical subtype. While the general estimates were consistent with expected ranges for mixed populations, high statistical heterogeneity was observed in almost all analyses, reflecting the variability between designs, diagnostic criteria, and geographical contexts. The meta-regression analysis by publication year showed no evidence of a significant temporal trend; however, when using the midpoint of the data collection period, a significant declining trend was identified, suggesting that the actual epidemiological period better captures temporal changes than the year of publication.

## 5 Heterogeneity and interpretation of pooled estimates

The high statistical heterogeneity observed across all analyses (*I*^2^ = 100%) is a consistent finding that warrants discussion. However, this result should be interpreted within the context of the broader methodological literature on meta-analyses of epidemiological rates. Recent evidence has demonstrated that extreme heterogeneity is an inherent characteristic of global prevalence and incidence meta-analyses. Migliavaca et al. (65) found a median *I*^2^ of 96.9% (IQR 90.5–98.7%) across 134 prevalence meta-analyses, and Vera-Ponce et al. (66), in an umbrella review of 53 global meta-analyses, documented *I*^2^ greater than or equal to 90% in 88.7% of studies and *I*^2^ > 99% in 64.2%. This variability reflects genuine differences between populations in age structure, cardiovascular risk profiles, diagnostic practices, healthcare access, and epidemiological surveillance systems. In this context, the random-effects model employed in our analysis provides an estimate of the average incidence across diverse settings rather than assuming a single common effect, which is the appropriate analytical framework when real between-study differences are expected (67, 68).

We conducted subgroup analyses by geographic region, publication decade, sampling type, sex, age, and HF phenotype, as well as meta-regressions by publication year, study midpoint, sample size, follow-up duration, diagnostic criteria type, data source, study design, and follow-up time measure type (Supplementary Material 8). Of these, the study midpoint was the strongest predictor of incidence (*p* = 0.0002, *R*^2^ = 23.75%), followed by sample size (*p* = 0.006, *R*^2^ = 13.79%) and follow-up duration (*p* = 0.039, *R*^2^ = 7.35%). Diagnostic criteria type, data source, and study design did not significantly explain the observed heterogeneity. None of these analyses substantially reduced heterogeneity below 99%, a result that is consistent with the expected behavior in epidemiological meta-analyses where multiple sources of variability operate simultaneously and cannot be fully captured by study-level moderators. Achieving meaningful reductions in heterogeneity in global incidence meta-analyses would likely require a degree of methodological standardization across primary studies (in diagnostic criteria, data sources, and population definitions) that does not currently exist in the HF literature. Until such standardization is achieved, the pooled estimates should be interpreted as weighted averages across heterogeneous contexts, with the subgroup-specific estimates providing more actionable information for regional health planning.

## 6 Comparison with previous literature

Our findings on the global incidence of HF, with a combined rate of 2.72 cases per 1,000 person-years (95% CI: 1.95–3.81), are generally consistent with the estimates reported in previous reviews. The study conducted by Conrad et al. (35) found incidence rates ranging between 2.1 and 3.9 per 1,000 person-years in European studies, similar to our estimate for this region (2.65 per 1,000 person-years). It also coincides with the systematic review by Savarese and Lund (2), who reported that the incidence of HF in the general population varies between 1 and 4 cases per 1,000 person-years, with our estimate falling within this interval. However, our review offers a more precise and updated estimate, incorporating studies published up to 2025.

The Global Burden of Disease Study 2019 reported a sustained increase in the absolute burden of HF worldwide, with approximately 65 million prevalent cases and an age-adjusted incidence of 4.2 per 1,000 inhabitants Roth et al. (69), higher than our estimate. This difference could be explained by the different methods employed, as the GBD uses complex statistical models that integrate multiple data sources, while our meta-analysis is based exclusively on published observational studies. It is important to note that the Heart Disease and Stroke Statistics Update 2022 from the American Heart Association estimated that approximately 1 million new cases of HF are diagnosed annually in the United States Tsao et al. (6), which, considering the U.S. adult population, translates to rates close to 5 per 1,000 person-years, consistent with our estimate for North America (6.06 per 1,000 person-years).

The regional differences identified in our analysis deserve special attention. The significantly higher incidence rate in North America compared to Europe coincides with the patterns reported by Ziaeian and Fonarow (70), who documented a higher burden of HF in the United States than in European countries. These differences could be attributed to the higher prevalence of cardiovascular risk factors in the North American population, particularly obesity and diabetes. According to NHANES 2017–2018 data, the prevalence of obesity in U.S. adults reached 42.4% Hales et al. (71), while in Europe it is around 23% according to the World Health Organization WHO Regional Office for Europe (72). Additionally, differences in epidemiological surveillance systems and diagnostic criteria could contribute to this variation, as described by Lippi and Sanchis-Gomar (4) in their review on comparative epidemiology of HF.

The incidence found in Asia (4.08 per 1,000 person-years) deserves particular analysis, as it is higher than historically reported for this region. The ASIAN-HF registry, which included data from 11 Asian countries, has documented an accelerated increase in the incidence of HF in these territories, especially in industrialized urban areas Lam et al. (73). A recent study based on the Korean National Health Insurance Service found that the annual incidence of HF increased from 0.7 to 9.7 per 1,000 people between 2002 and 2018 Lee et al. (74), consistent with our estimate for South Korea (9.87 per 1,000 person-years). The rapid epidemiological transition, population aging, and westernization of lifestyles in Asia could explain this emerging phenomenon, as proposed by the analysis of Zhang et al. (75) on the changing epidemiology of HF in China.

The sex differences identified in our meta-analysis, with slightly higher rates in men (1.93 vs. 1.62 per 1,000 person-years in women), are consistent with the findings of the ARIC (Atherosclerosis Risk in Communities) study, which reported a 5-year cumulative incidence of 15.7 vs. 13.3 per 1,000 in men and women respectively Loehr et al. (28). However, the difference observed in our review is less pronounced than that reported in previous studies such as Roger et al. (76), who found an age-adjusted incidence of 3.78 vs. 2.89 per 1,000 person-years for men and women in Olmsted County. This apparent convergence could reflect an important epidemiological change: while rates in men have tended to stabilize or even decrease in some contexts due to improvements in the management of ischemic heart disease, rates in women have not shown such a marked decrease, as documented by Shah et al. (10) in an analysis of trends from the Get With The Guidelines-Heart Failure Registry.

The pronounced increase in incidence in the population over 50 years (12.38 vs. 3.05 per 1,000 person-years in the general population) is in line with the findings of the Framingham study, which documented an exponential increase in HF risk with age. Levy et al. (77) found that the incidence of HF increased from 3 per 1,000 person-years at 50–59 years to 27 per 1,000 person-years at 80–89 years in men, with a similar pattern in women. More recent data from the Copenhagen General Population Study confirmed this age gradient, with an approximately 8-fold increase in HF incidence between 50 and 90 years (78). Our results confirm the persistence of this pattern in contemporary studies and diverse geographical contexts.

Our estimates for specific HF subtypes (1.77 for HFrEF and 1.58 for HFpEF per 1,000 person-years) differ from those reported by Gerber et al. (80), who found in the Olmsted County cohort a higher incidence of HFpEF (2.5 vs. 1.9 per 1,000 person-years for HFrEF). This discrepancy should be interpreted with caution given that only four studies in our meta-analysis reported specific incidence by subtype, resulting in wide confidence intervals (HFpEF: 0.41–6.06; HFrEF: 0.56–5.60) that reflect substantial imprecision. These results, while providing a quantitative reference point for an important clinical question, are underpowered and should not be used as definitive estimates. Additionally, the classification of HF phenotypes has evolved significantly over the study period. Earlier studies used a binary classification based on ejection fraction (reduced vs. preserved), while current guidelines, including the 2021 ESC update (1), recognize a three-tier system that includes HF with mildly reduced ejection fraction (HFmrEF, LVEF 41%–49%). None of the included studies reported incidence data specifically for HFmrEF, precluding analysis of this increasingly recognized category. The variable cutoff points for ejection fraction used across studies and time periods further complicate between-study comparisons and contribute to the heterogeneity observed in our phenotype-specific analysis.

Several factors, including the inclusion in our analysis of a broader representation of geographical regions and the methodological heterogeneity between studies, could explain the absence of a clear temporal trend in our meta-regression by publication year. However, the significant declining trend observed when using the midpoint of data collection (*p* = 0.0002) suggests that HF incidence may indeed be decreasing over calendar time, but this effect is obscured when publication year is used as a proxy, given the variable lag between data collection and publication. Furthermore, as proposed by Sidney et al. (79), the counteracting effects of improvements in primary prevention (which would reduce incidence) and population aging, together with increased post-infarction survival (which would increase it), could result in a net effect of apparent stability in crude rates.

## 7 Implications for public health and global health

The estimates of HF incidence obtained in this meta-analysis have important implications for global healthcare systems. The combined rate of 2.72 cases per 1,000 person-years, which increases to 12.38 per 1,000 person-years in the population over 50 years, suggests that health planning must contemplate a sustained increase in healthcare demand related to HF, especially in countries with aging populations. The data indicate that the economic impact will be considerable, given that each HF hospitalization represents a significant cost for healthcare systems, not counting the indirect costs associated with productivity loss and informal care. These findings point to the urgent need to redirect resources toward structured primary prevention programs specifically targeting at-risk populations and toward integrated care models that reduce avoidable hospitalizations, particularly in resource-limited settings where the proportional impact of HF could be even greater.

The marked regional disparities identified in our study, with higher rates in North America (6.06) than in Europe (2.65 per 1,000 person-years), reveal the need to adapt public health strategies to local contexts. The accelerated increase in HF incidence in Asia (4.08 per 1,000 person-years) suggests that countries in epidemiological transition require specific policies that simultaneously address traditional and emerging cardiac diseases. The data indicate that these regions could benefit from differentiated interventions, with greater emphasis on controlling emerging risk factors such as obesity and diabetes in North America, while in Asia it would be a priority to develop early detection infrastructures given the rapid increase observed. Our results provide the necessary epidemiological basis to guide resource distribution in global health initiatives and adapt interventions to the epidemiological characteristics of each region.

The incidence differences identified between men (1.93) and women (1.62 per 1,000 person-years), although less pronounced than in previous studies, reflect the persistence of gender disparities that require specific attention in health policies. The results suggest that there could be differences in both exposure to risk factors and access to diagnosis between sexes. The narrower gap observed compared to historical studies could be interpreted as an advance in diagnostic equity, but also as a possible relative increase in incidence in women associated with changes in cardiovascular risk patterns. These findings support the need to implement screening programs that consider gender-specific differences and prevention strategies adapted to the different risk profiles and clinical presentations between men and women, an aspect frequently neglected in current clinical practice guidelines.

The growing burden of heart failure with preserved ejection fraction (HFpEF) documented in our study, with an incidence of 1.58 per 1,000 person-years (similar to that of HF with reduced ejection fraction), poses a particular challenge for healthcare systems. This finding is especially relevant considering the scarcity of specific treatments with solid evidence for this phenotype, which implies that a growing proportion of patients with HF will receive suboptimal therapeutic approaches. The data suggest the urgent need to redirect research resources toward this frequently neglected phenotype, as well as to develop specific care programs that focus on the comprehensive management of comorbidities, frequently present in these patients. The convergence in incidence between both phenotypes also calls attention to the importance of improving the training of healthcare professionals in the recognition and management of HFpEF, traditionally underdiagnosed in primary care settings.

Finally, the absence of a clear temporal trend by publication year, despite advances in primary cardiovascular prevention during the last decades, alongside the significant declining trend by data collection midpoint, suggests a complex pattern where improvements in prevention may be partially counteracted by population aging and increased survival after acute cardiovascular events. This balance has profound implications for the sustainability of healthcare systems, indicating the need for a dual approach: on one hand, intensifying primordial prevention strategies focused on modifiable risk factors from early ages, and on the other, developing care models that optimize resources in a scenario of increasing demand. The results of our study suggest that public health policies should be reoriented toward a complete life-cycle approach to HF, from early prevention to optimized chronic management, with special attention to transitions between levels of care, a critical point where discontinuities that increase readmissions and costs associated with this pathology frequently occur.

## 8 Strengths and limitations

Our study presents important methodological strengths that reinforce the validity of the findings. We conducted an exhaustive and systematic literature search in multiple databases without language restrictions, identifying 42 studies (2001–2025) with clearly defined eligibility criteria. Risk of bias was assessed using the Munn tool, with 57.1% of studies classified as low risk and no study classified as high risk (Supplementary Material S9). We implemented robust statistical methods adapted to the expected heterogeneity, including logarithmic transformation of reported incidence rates with Poisson-derived variance estimation, random-effects models with REML, and comprehensive sensitivity analyses comparing five different variance estimators, all of which produced convergent results (Supplementary Material S7). We performed stratified analyses by region, sex, age, specific phenotypes, diagnostic criteria type, data source, study design, and risk of bias, as well as meta-regressions by eight different moderators (Supplementary Material S8). The broad geographical coverage is particularly worth noting, with studies from 18 different countries across three continents, including both urban and rural settings, and the consideration of various diagnostic criteria (clinical, administrative, and mixed) that reflects the complexity of contemporary epidemiological practice.

Despite its strengths, our study has limitations that should be considered when interpreting the results. The marked statistical heterogeneity (*I*^2^ = 100%) persists even in stratified analyses, reflecting substantial methodological differences between primary studies, although this level is consistent with similar global meta-analyses (65, 66). There is a geographical underrepresentation, with absence of studies from Africa, Oceania, and Latin America and predominance of research from high-income countries, which limits generalization to regions where HF epidemiological profiles might differ significantly; the term “global” in our title refers to the worldwide scope of our search strategy, conducted without geographical or language restrictions, and this uneven distribution reflects a gap in the primary literature rather than in our methodology. The variability in diagnostic criteria introduces complexity in comparability: validation studies have reported positive predictive values for ICD-based HF identification ranging from 70% to 95%, with administrative codes tending to capture more severe or hospitalized cases while adjudicated clinical criteria may have higher sensitivity for early-stage disease, although our meta-regression showed that diagnostic criteria type did not significantly influence the pooled estimates (*p* = 0.323). The age stratification was limited to a dichotomous classification (under and over 50 years), as only four studies exclusively evaluated older populations and no study provided separate data for the very elderly (over 80 years); this reflects how HF incidence is reported in the current literature rather than an analytical choice. The phenotype-specific analysis (HFrEF and HFpEF) was based on only four studies, resulting in wide confidence intervals that reflect substantial imprecision, and HF with mildly reduced ejection fraction (HFmrEF), recognized in the 2021 ESC guidelines, could not be analyzed as no study reported incidence data for this category.

Several additional limitations, inherent to the primary studies, should be acknowledged. Our country- and region-level estimates are subject to ecological fallacy, as they do not capture within-country heterogeneity related to urban-rural differences and socioeconomic disparities. Reported incidence rates partly reflect diagnostic capacity and healthcare access rather than true disease occurrence alone, and our estimates may be affected by survivor bias, as individuals who die before receiving a formal HF diagnosis would not be captured as incident cases. Diagnostic criteria for HF evolved substantially during the study period (2000–2025), from predominantly clinical definitions to frameworks incorporating biomarkers and imaging, and ICD coding transitioned from ICD-9 to ICD-10 in many countries, which may affect temporal comparisons. Our analysis could not directly evaluate the influence of comorbidities such as diabetes and obesity on regional patterns, as these data were inconsistently reported across primary studies; future meta-analyses incorporating individual participant data would be better positioned to address this question. The true global burden of HF may differ substantially from our estimates, particularly in low- and middle-income regions where access to diagnostic services and population age structure differ considerably from those of the countries represented in this review, reinforcing the urgent need for population-based incidence studies in underrepresented regions.

## 9 Conclusions

Our meta-analysis provides an updated and methodologically rigorous estimate of the global incidence of heart failure, establishing a combined rate of 2.72 cases per 1,000 person-years (95% CI: 1.95–3.81) in the general population, with variations according to geographic region, sex, age, and phenotype. The findings show a particularly high burden in North America and Asia, a pronounced increase in the population over 50 years, and a progressive convergence between subtypes with reduced and preserved ejection fraction. Although no significant temporal trend was observed by publication year, the meta-regression by data collection midpoint identified a significant declining trend in HF incidence over calendar time (*p* = 0.0002), suggesting that advances in cardiovascular prevention may be having a measurable effect that is masked when publication year is used as the temporal reference. This scenario configures heart failure as a persistent and growing global health challenge, requiring differentiated strategies according to regional and sociodemographic context, with special attention to aging populations where the incidence quadruples compared to the general population. However, the pooled estimate is predominantly derived from high-income countries, and the absence of data from Africa, Latin America, and Oceania limits the generalizability of these findings to low- and middle-income settings.

Based on the findings of this study, we recommend implementing comprehensive epidemiological surveillance programs for heart failure with standardized methodology, particularly in underrepresented regions (Africa, Latin America, Oceania), allowing the identification of specific patterns and more precise monitoring of temporal trends. It is a priority to develop differentiated preventive strategies according to the regional epidemiological profile, with emphasis on obesity and diabetes control in North America, and on early detection in Asian urban areas with rapidly increasing incidence. Healthcare systems must prepare for increasing demand through care models that optimize resources and reduce avoidable hospitalizations, especially in primary care and transitions between levels of care. Future research should prioritize studies on heart failure with preserved ejection fraction, whose incidence comparable to the reduced form contrasts with its lower pathophysiological understanding and limited therapeutic options. Finally, we recommend systematically incorporating gender perspectives in prevention, diagnosis, and treatment, adapting interventions to the different risk profiles and clinical presentation between sexes, and paying special attention to the older female population, where the most determinant risk factors identified in our analysis converge.

## Data Availability

The original contributions presented in the study are included in the article/Supplementary Material, further inquiries can be directed to the corresponding author.
