# Supplementary material for: Global heart failure incidence rate: an updated systematic review and meta-analysis
Source: Front Cardiovasc Med. 2026 Mar 20;13:1714070. doi: 10.3389/fcvm.2026.1714070 (PMC13046512; doi:10.3389/fcvm.2026.1714070)
Supplement: Supplementary file 1 [file Datasheet1.docx]

Supplementary Material

**
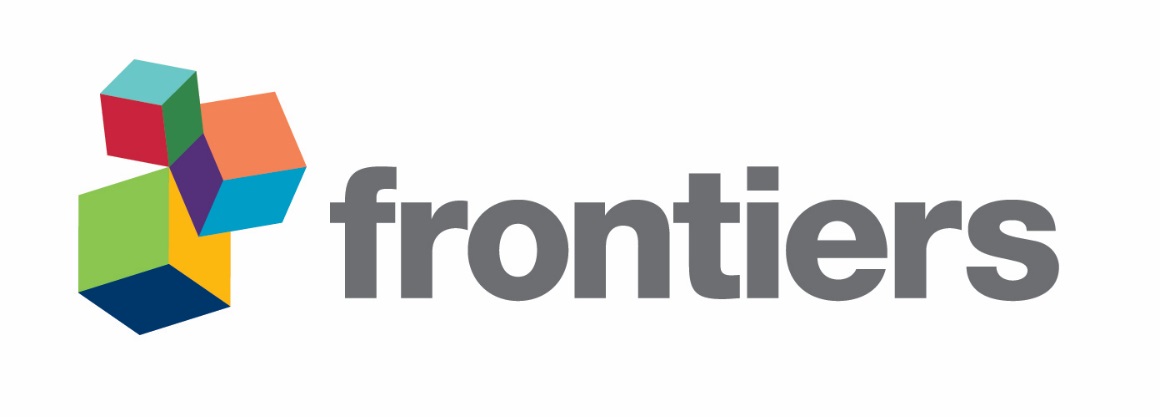
**

**Supplementary material 1.** PRISMA 2020 Checklist

**Supplementary material 2.** Search strategy

**Supplementary material 3.** Summary of the characteristics of the selected articles

**Supplementary Material 4**. Funnel Plot of HF Incidence Rates

**Supplementary material 1**. PRISMA 2020 Checklist

| **Section and Topic** | **Item #** | **Checklist item** |
| --- | --- | --- |
| **TITLE** | | |
| Title | 1 | Identify the report as a systematic review. |
| **ABSTRACT** | | |
| Abstract | 2 | See the PRISMA 2020 for Abstracts checklist. |
| **INTRODUCTION** | | |
| Rationale | 3 | Describe the rationale for the review in the context of existing knowledge. |
| Objectives | 4 | Provide an explicit statement of the objective(s) or question(s) the review addresses. |
| **METHODS** | | |
| Eligibility criteria | 5 | Specify the inclusion and exclusion criteria for the review and how studies were grouped for the syntheses. |
| Information sources | 6 | Specify all databases, registers, websites, organisations, reference lists and other sources searched or consulted to identify studies. Specify the date when each source was last searched or consulted. |
| Search strategy | 7 | Present the full search strategies for all databases, registers and websites, including any filters and limits used. |
| Selection process | 8 | Specify the methods used to decide whether a study met the inclusion criteria of the review, including how many reviewers screened each record and each report retrieved, whether they worked independently, and if applicable, details of automation tools used in the process. |
| Data collection process | 9 | Specify the methods used to collect data from reports, including how many reviewers collected data from each report, whether they worked independently, any processes for obtaining or confirming data from study investigators, and if applicable, details of automation tools used in the process. |
| Data items | 10a | List and define all outcomes for which data were sought. Specify whether all results that were compatible with each outcome domain in each study were sought (e.g. for all measures, time points, analyses), and if not, the methods used to decide which results to collect. |
|  | 10b | List and define all other variables for which data were sought (e.g. participant and intervention characteristics, funding sources). Describe any assumptions made about any missing or unclear information. |
| Study risk of bias assessment | 11 | Specify the methods used to assess risk of bias in the included studies, including details of the tool(s) used, how many reviewers assessed each study and whether they worked independently, and if applicable, details of automation tools used in the process. |
| Effect measures | 12 | Specify for each outcome the effect measure(s) (e.g. risk ratio, mean difference) used in the synthesis or presentation of results. |
| Synthesis methods | 13a | Describe the processes used to decide which studies were eligible for each synthesis (e.g. tabulating the study intervention characteristics and comparing against the planned groups for each synthesis (item #5)). |
|  | 13b | Describe any methods required to prepare the data for presentation or synthesis, such as handling of missing summary statistics, or data conversions. |
|  | 13c | Describe any methods used to tabulate or visually display results of individual studies and syntheses. |
|  | 13d | Describe any methods used to synthesize results and provide a rationale for the choice(s). If meta-analysis was performed, describe the model(s), method(s) to identify the presence and extent of statistical heterogeneity, and software package(s) used. |
|  | 13e | Describe any methods used to explore possible causes of heterogeneity among study results (e.g. subgroup analysis, meta-regression). |
|  | 13f | Describe any sensitivity analyses conducted to assess robustness of the synthesized results. |
| Reporting bias assessment | 14 | Describe any methods used to assess risk of bias due to missing results in a synthesis (arising from reporting biases). |
| Certainty assessment | 15 | Describe any methods used to assess certainty (or confidence) in the body of evidence for an outcome. |
| **RESULTS** | | |
| Study selection | 16a | Describe the results of the search and selection process, from the number of records identified in the search to the number of studies included in the review, ideally using a flow diagram. |
|  | 16b | Cite studies that might appear to meet the inclusion criteria, but which were excluded, and explain why they were excluded. |
| Study characteristics | 17 | Cite each included study and present its characteristics. |
| Risk of bias in studies | 18 | Present assessments of risk of bias for each included study. |
| Results of individual studies | 19 | For all outcomes, present, for each study: (a) summary statistics for each group (where appropriate) and (b) an effect estimate and its precision (e.g. confidence/credible interval), ideally using structured tables or plots. |
| Results of syntheses | 20a | For each synthesis, briefly summarise the characteristics and risk of bias among contributing studies. |
|  | 20b | Present results of all statistical syntheses conducted. If meta-analysis was done, present for each the summary estimate and its precision (e.g. confidence/credible interval) and measures of statistical heterogeneity. If comparing groups, describe the direction of the effect. |
|  | 20c | Present results of all investigations of possible causes of heterogeneity among study results. |
|  | 20d | Present results of all sensitivity analyses conducted to assess the robustness of the synthesized results. |
| Reporting biases | 21 | Present assessments of risk of bias due to missing results (arising from reporting biases) for each synthesis assessed. |
| Certainty of evidence | 22 | Present assessments of certainty (or confidence) in the body of evidence for each outcome assessed. |
| **DISCUSSION** | | |
| Discussion | 23a | Provide a general interpretation of the results in the context of other evidence. |
|  | 23b | Discuss any limitations of the evidence included in the review. |
|  | 23c | Discuss any limitations of the review processes used. |
|  | 23d | Discuss implications of the results for practice, policy, and future research. |
| **OTHER INFORMATION** | | |
| Registration and protocol | 24a | Provide registration information for the review, including register name and registration number, or state that the review was not registered. |
|  | 24b | Indicate where the review protocol can be accessed, or state that a protocol was not prepared. |
|  | 24c | Describe and explain any amendments to information provided at registration or in the protocol. |
| Support | 25 | Describe sources of financial or non-financial support for the review, and the role of the funders or sponsors in the review. |
| Competing interests | 26 | Declare any competing interests of review authors. |
| Availability of data, code and other materials | 27 | Report which of the following are publicly available and where they can be found: template data collection forms; data extracted from included studies; data used for all analyses; analytic code; any other materials used in the review. |

From: Page MJ, McKenzie JE, Bossuyt PM, Boutron I, Hoffmann TC, Mulrow CD, et al. The PRISMA 2020 statement: an updated guideline for reporting systematic reviews. BMJ 2021;372:n71. doi: 10.1136/bmj.n71. This work is licensed under CC BY 4.0. To view a copy of this license, visit <https://creativecommons.org/licenses/by/4.0/>

**Supplementary material 2**. Search strategy

**Search strategy in PUBMED**

| **Step** | **Strategy** |
| --- | --- |
| #1 | heart failure"[ti] OR "cardiac failure"[ti] OR "congestive heart failure"[ti] OR "insuficiencia cardiaca"[ti] OR "falla cardiaca"[ti] |
| #2 | incidence[tiab] OR "incidencia"[tiab] |
| #3 | #1 AND #2 |

**Search strategy in SCOPUS**

| **Step** | **Strategy** |
| --- | --- |
| #1 | TITLE ( "heart failure" OR "cardiac failure" OR "congestive heart failure" OR "insuficiencia cardiaca" OR "falla cardiaca" ) |
| #2 | TITLE-ABS ( incidence OR "incidencia" ) |
| #4 | #1 AND #2 |

**Search strategy in Web of Science**

| **Step** | **Strategy** |
| --- | --- |
| #1 | TI=( "heart failure" OR "cardiac failure" OR "congestive heart failure" OR "insuficiencia cardiaca" OR "falla cardiaca" ) |
| #2 | TS=( incidence OR "incidencia" ) |
| #4 | #1 AND #2 AND |

**Search strategy in EMBASE**

| **Step** | **Strategy** |
| --- | --- |
| #1 | ('heart failure':ti OR 'cardiac failure':ti OR 'congestive heart failure':ti OR 'insuficiencia cardiaca':ti OR 'falla cardiaca':ti) |
| #2 | (incidence:ab,ti OR 'incidencia':ab,ti) |
| #3 | #1 AND #2 |

**Supplementary material 3.** Summary of the characteristics of the selected articles

| **Author** | **Year** | **Country** | **Study Type** | **Sampling** | **Sample Size** | **Follow-up Years** | **Mean Age (Baseline/Event)** | **Diagnostic Criteria** |
| --- | --- | --- | --- | --- | --- | --- | --- | --- |
| Fox | 2001 | United Kingdom | Prospective observational study | Non-probabilistic | 292,000 | 1.25 years (15 months) | Median event: 76 years (M 75, F 79) | Clinical diagnosis + signs + echo (ESC guideline) |
| Bleumink | 2004 | Netherlands | Prospective population cohort (Rotterdam Study) | Non-probabilistic | 7,734 | Median: 7.1 years (IQR 5.7-8.0) | Baseline: 70.4 ± 9.7 years; Event: M 77.5, F 82.5 | ESC: symptoms + objective evidence (X-ray, echo) + cardiologist validation |
| Murphy | 2004 | Scotland | CMR cohort (Continuous Morbidity Recording) | Non-probabilistic | 305,555 | 1 year (April 1999-March 2000) | Not reported | Clinical diagnosis (Read codes) |
| De Giuli | 2005 | United Kingdom | Retrospective cohort (GPRD) | Probabilistic | 696,884 | 3 years (1991-1994) | Diagnosis mean: 77 years (M 75±9, F 79±9) | Clinical diagnosis by a general physician/hospital letter |
| Van Jaarsveld | 2005 | Netherlands | Longitudinal cohort (GLAS) | Probabilistic | 5,279 | 5 years (mean: 28 months) | HF diagnosis: mean 75.5 years | Clinical diagnosis: ≥3 of 5 clinical criteria (edema, dyspnea, etc.) |
| Loehr | 2008 | USA | Prospective population cohort (ARIC) | Probabilistic | 14,994 | 13 years (1987-2002) | Baseline: 54 years; Event: 65.4 years | ICD-9: 428.x; ICD-10: I50 by hospitalization or death |
| Borne | 2012 | Sweden | Population cohort (Malmo Diet and Cancer Study) | Non-probabilistic | 26,653 | Mean: 14 years | Baseline: 58 years | Hospital diagnosis (ICD-8, 9, 10) |
| Rautiainen | 2013 | Sweden | Prospective cohort (Swedish Mammography Cohort) | Probabilistic | 33,713 | Median: 11.3 years (394,059 person-years) | Baseline: 61.2 years | Hospital or death diagnosis, ESC validated |
| Shah | 2013 | USA | Cohort (MESA) | Probabilistic | 4,652 | Median: 7.6 years | Baseline: 61.2 years | Adjudicated clinical diagnosis (WHI and CHS) |
| Zarrinkoub | 2013 | Sweden | Population cross-sectional study (VAL, Stockholm) | Probabilistic | 2.1 million | 1997-2010 | Diagnosis 2010: 77 years (M 74±13, F 80±12) | Diagnosis in clinical registries ICD-10 |
| Ohlmeier | 2015 | Germany | Retrospective cohort (GePaRD) | Non-probabilistic | 6,284,194 | 1 year (2006) | Baseline: 39 years (population); Event: 71.9 years (cases) | Diagnosis ICD-10-GM + antihypertensive medication |
| Nayor | 2016 | USA | Longitudinal cohort (Framingham Offspring Study) | Probabilistic | 3,201 | Mean: 12.3 years | Baseline: 59 years | Framingham criteria; EF by echocardiogram |
| Conrad | 2017 | United Kingdom | Retrospective population cohort (CPRD-HES) | Non-probabilistic | 3,992,417 | 13 years (2002-2014) | Mean event: 76.7 years (M 74.0, F 79.4) | Electronic diagnosis (Read, ICD-10) in primary care and hospital |
| Piccinni | 2017 | Italy | Cross-sectional and cohort study (HSD) | Non-probabilistic | 1,080,102 | 1 year (2013) | Not reported | Clinical diagnosis (ICD-9-CM: 428., 402., 404.*) |
| Störk | 2017 | Germany | Retrospective cohort (Health Risk Institute) | Non-probabilistic | 3,132,337 | 1 year (follow-up per person) | Mean: 76.2 years | Diagnosis ICD-10-GM (not clinically validated) |
| Hinton | 2018 | United Kingdom | Cross-sectional and follow-up study (RCGP RSC) | Non-probabilistic | 1,275,174 | 5 years (2012-2016) | Total mean age: 47.5 years | Coded clinical diagnosis (Read codes) |
| Tsao | 2018 | USA | Cohort (FHS + CHS) | Probabilistic | 6,455 | 10 years (2000-2009) | Baseline: 74 ± 9 years | Clinical diagnosis confirmed by panel (FHS and CHS criteria) |
| Huusko | 2019 | Finland | Retrospective hospital study + biobank | Non-probabilistic | 500,000 | Maximum 9 years (2004-2013) | Total mean: 77.1 ± 11.3 years | Diagnosis ICD-10 I50 in electronic medical record |
| Magnussen | 2019 | Europe multicentric | Prospective cohort (BiomarCaRE) | Non-probabilistic | 78,657 | Median: 12.7 years (range 0-29) | Baseline median: 49.4 years (M 49.9, F 49.0) | Diagnosis hospital registries, deaths, self-report (ICD-10) |
| Uijl | 2019 | Netherlands | Prospective cohort (EPIC-NL) | Non-probabilistic | 37,803 | Median: 15.2 years (IQR 14.1-16.5) | Baseline: 49.4 ± 11.9 years | Diagnosis hospital registries (ICD-9 and ICD-10) |
| Bergsten | 2019 | USA | Longitudinal population cohort (NHEFS) | Probabilistic | 14,407 | 20 years (1971-1992) | Not reported | Diagnosis by ICD-9 codes in hospitalization or death |
| Sillars | 2019 | United Kingdom | Prospective cohort (UK Biobank) | Probabilistic | 468,941 | Mean: 8.2 years | M: 56.2 years, F: 56.1 years | Hospital diagnosis ICD-10 (I50.0, I50.2, I50.9) |
| Ergatoudes | 2019 | Sweden | Longitudinal cohort | Probabilistic | 1,652 | 21 years (both cohorts) | Baseline: 50 years; Event: 71 years | Hospitalization or death by HF (ICD-8, ICD-9, ICD-10) |
| Bai | 2020 | Canada (Toronto) | Retrospective population cohort (ONPHEC) | Probabilistic | 986,295 | 15 years (2001-2015) | Baseline: 55.6 years | Diagnosis in administrative database ICD-9/ICD-10 |
| Tun | 2020 | USA | Prospective cohort (Framingham Offspring Study) | Probabilistic | 3,416 | Median: 18 years | Baseline: 59 ± 10 years | Diagnosis FHS committee; subtypes HFrEF, HFpEF by EF% |
| Lewis | 2020 | USA | Population cohort (ARIC, DHS, MESA) | Probabilistic | 15,710 | 10 years | Baseline: 57 years | Hospital diagnosis ICD-9 or outpatient |
| Ødegaard | 2020 | Norway | Population cohort (NorPD) | Probabilistic | 3,975,642 | 1 year | Not reported | Clinical diagnosis from prescriptions ICD-10 (I50, I11, I13, I42) |
| Yan | 2021 | USA | Multicentric cohort (SHHS) | Probabilistic | 4,887 | Mean: 10.4 ± 3.3 years | Baseline: 63.6 ± 11.0 years | Adjudicated diagnosis (symptoms + imaging) |
| Fujimoto | 2021 | Japan | Retrospective population cohort (KUNIUMI Registry) | Probabilistic | 134,774 | 3 years (2015-2017) | Baseline: 82.1 ±11.5 years | Framingham diagnosis + JCS/JHFS 2017 guidelines; HFrEF and HFpEF by EF |
| Wang | 2021 | China | Retrospective cohort (national urban insurance database) | Probabilistic | 49,572,122 | 1 year (2017) | Baseline: 63.9 years (M: 64.2, F: 63.7) | Diagnosis ICD-10 (I50.x, I11, I13, etc.) |
| Guardino | 2022 | USA | Prospective cohort (Framingham Offspring Study) | Probabilistic | 3,412 | Median: 10.3 years | Baseline: 67 years | Framingham criteria: ≥2 major or ≥1 major and ≥2 minor |
| Ødegaard | 2022 | Norway | Retrospective cohort (National Registry) | Probabilistic | 186,297 | 5 years (2014-2018) | Median: 79 years (M: 75, F: 83) | Validated ICD-10 diagnosis in hospitalization and outpatient |
| Zhu | 2022 | United Kingdom | Prospective cohort (UK Biobank) | Probabilistic | 464,483 | Median: 10.1 years | Baseline: 57 years | Hospital diagnosis ICD-10 (I50) |
| Fu | 2023 | China | Community cohort (Guangzhou) | Probabilistic | 2,597 | 3 years | Baseline: 65 years | Clinical diagnosis ICD-10 and specialist evaluation |
| Bellanca | 2023 | England | Non-interventional cohort (CPRD Aurum + HES + ONS) | Probabilistic | 11,210,522 | Up to 5 years (2015-2019) | Baseline: 75 years; HFrEF: 72, HFpEF: 71.7 | Diagnosis CPRD/HES, recorded EF; expert review |
| Vainshelboim | 2023 | USA | Prospective cohort (VETS) | Non-probabilistic | 5,539 | Mean: 14.3 years (range 6-18) | Baseline: 59 ±11.5 years; Event: 62.6±10.9 years | VA medical diagnosis; ICD-9: 428.0-428.43 |
| Zhao | 2024 | United Kingdom | Prospective cohort (UK Biobank) | Probabilistic | 274,608 | Median: 13.5 years | Baseline: 56.5 years; Event: 62.1 years | Hospital diagnosis ICD-9/ICD-10 (I50) |
| Wang | 2024 | United Kingdom | Prospective cohort (UK Biobank) | Probabilistic | 330,362 | Median: 14.1 years | Event: 61.8 years | Diagnosis by ICD-10 in hospital registries, deaths, and self-reports |
| Chang | 2025 | South Korea | Longitudinal population cohort (NHIS-HEALS) | Probabilistic | 293,968 | Median: 9.6 years (IQR 9.2-10.13) | Not reported | Diagnosis ICD-10: I50 in national insurance registries |
| Hu | 2025 | United Kingdom | Prospective cohort (UK Biobank) | Probabilistic | 336,939 | Median: 13.6 years (IQR 12.91-14.25) | Baseline: 56.1 years | Diagnosis ICD-10: I50 (hospitals, primary care, deaths, verified self-report) |
| Wang | 2025 | United Kingdom | Prospective cohort (UK Biobank) | Probabilistic | 236,754 | Median: 13.61 years | Baseline: 55.8 years; Event: 61.7 ±6.2 years | Hospital diagnosis ICD-10: I50.0, I50.1, I50.9 |
| Panchal | 2025 | England | Retrospective cohort (CPRD GOLD and Aurum + HES + ONS) | Probabilistic | 735,810 | Median: 10 years (IQR 5.3-10) | Not reported | Clinical diagnosis CPRD/HES/ONS; ischemic HF if IHD posterior |

*Note: M = Male, F = Female, IQR = Interquartile Range, EF = Ejection Fraction, HFrEF = Heart Failure with reduced Ejection Fraction, HFpEF = Heart Failure with preserved Ejection Fraction, IHD = Ischemic Heart Disease*

**Supplementary Material 4**. Risk of bias assessment


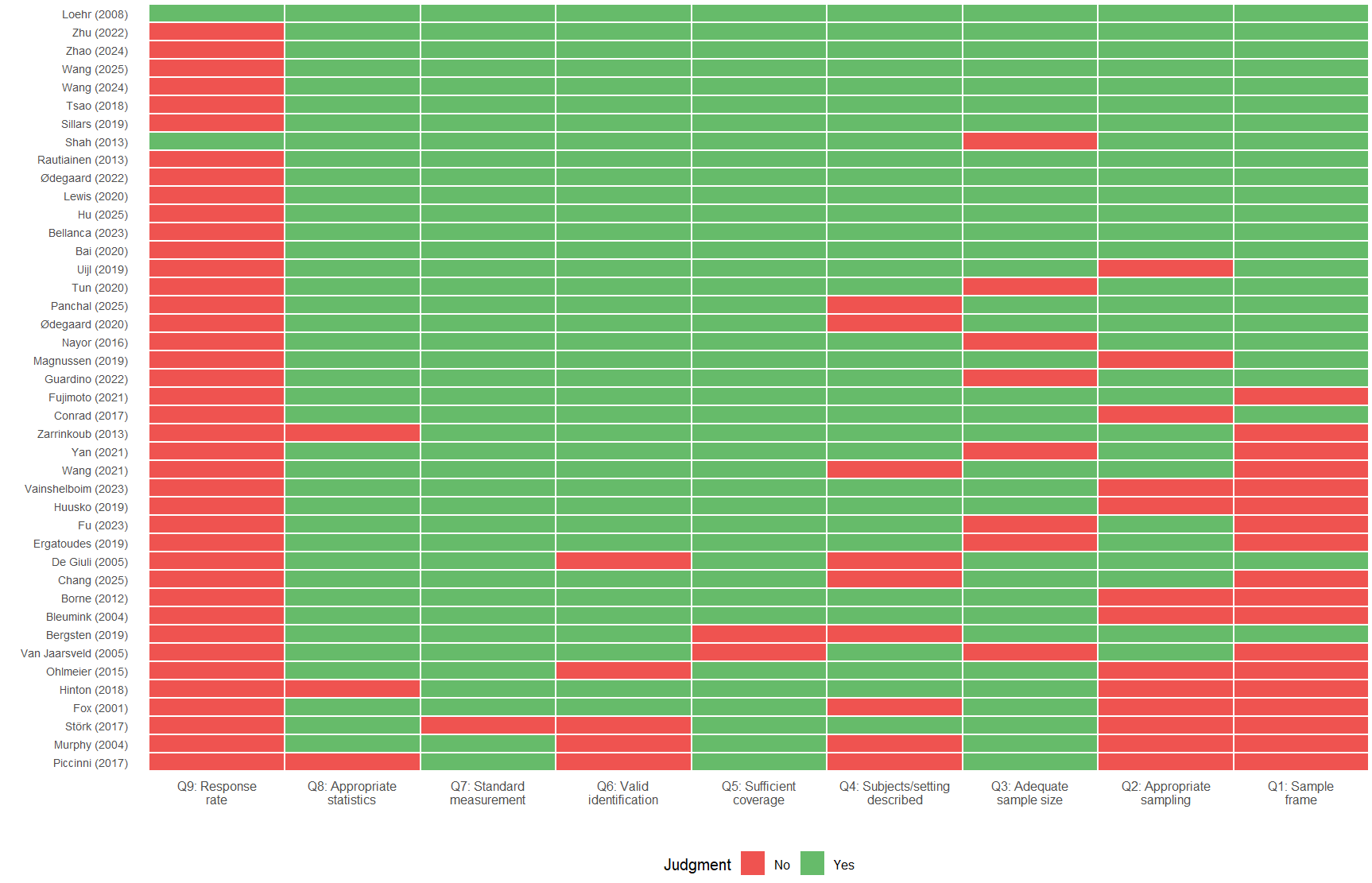


**Supplementary Material 5**. Meta-regression analysis

| **Moderator** | **Type** | **N** | **QM** | **p-value** | **R² (%)** | **Interpretation** |
| --- | --- | --- | --- | --- | --- | --- |
| Publication year | Continuous | 42 | 1.30 | 0.255 | 0.72 | NS |
| Study midpoint | Continuous | 42 | 13.76 | 0.0002 | 23.75 | Significant |
| Diagnostic criteria | Categorical | 42 | 2.26 | 0.323 | 0.65 | NS |
| Data source | Categorical | 42 | 1.30 | 0.254 | 0.75 | NS |
| Study design | Categorical | 42 | 0.44 | 0.801 | 0.00 | NS |
| Sample size (log) | Continuous | 42 | 7.53 | 0.006 | 13.79 | Significant |
| Follow-up duration | Continuous | 42 | 4.26 | 0.039 | 7.35 | Significant |
| Time measure type | Categorical | 42 | 0.65 | 0.885 | 0.00 | NS |


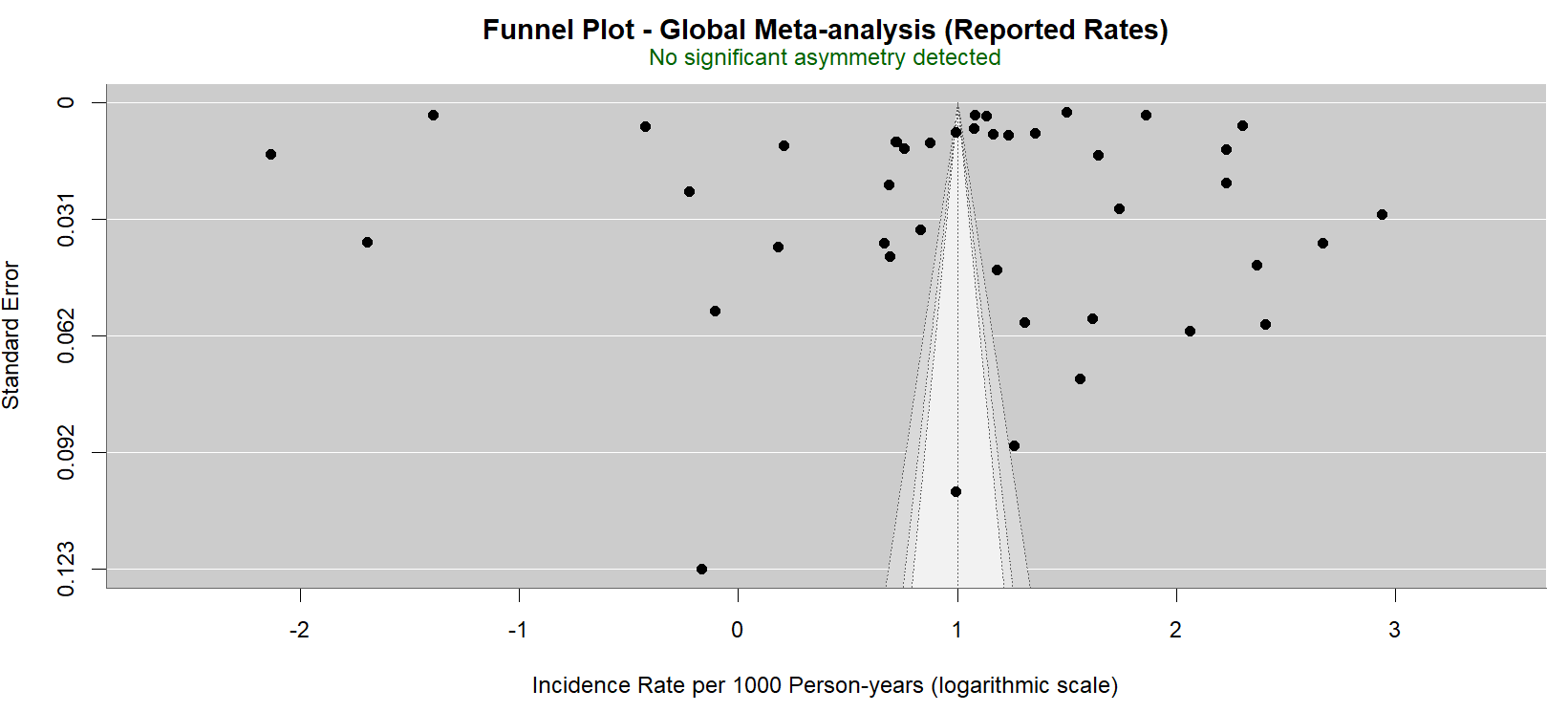


**Supplementary Material 6**. Funnel Plot of HF Incidence Rates


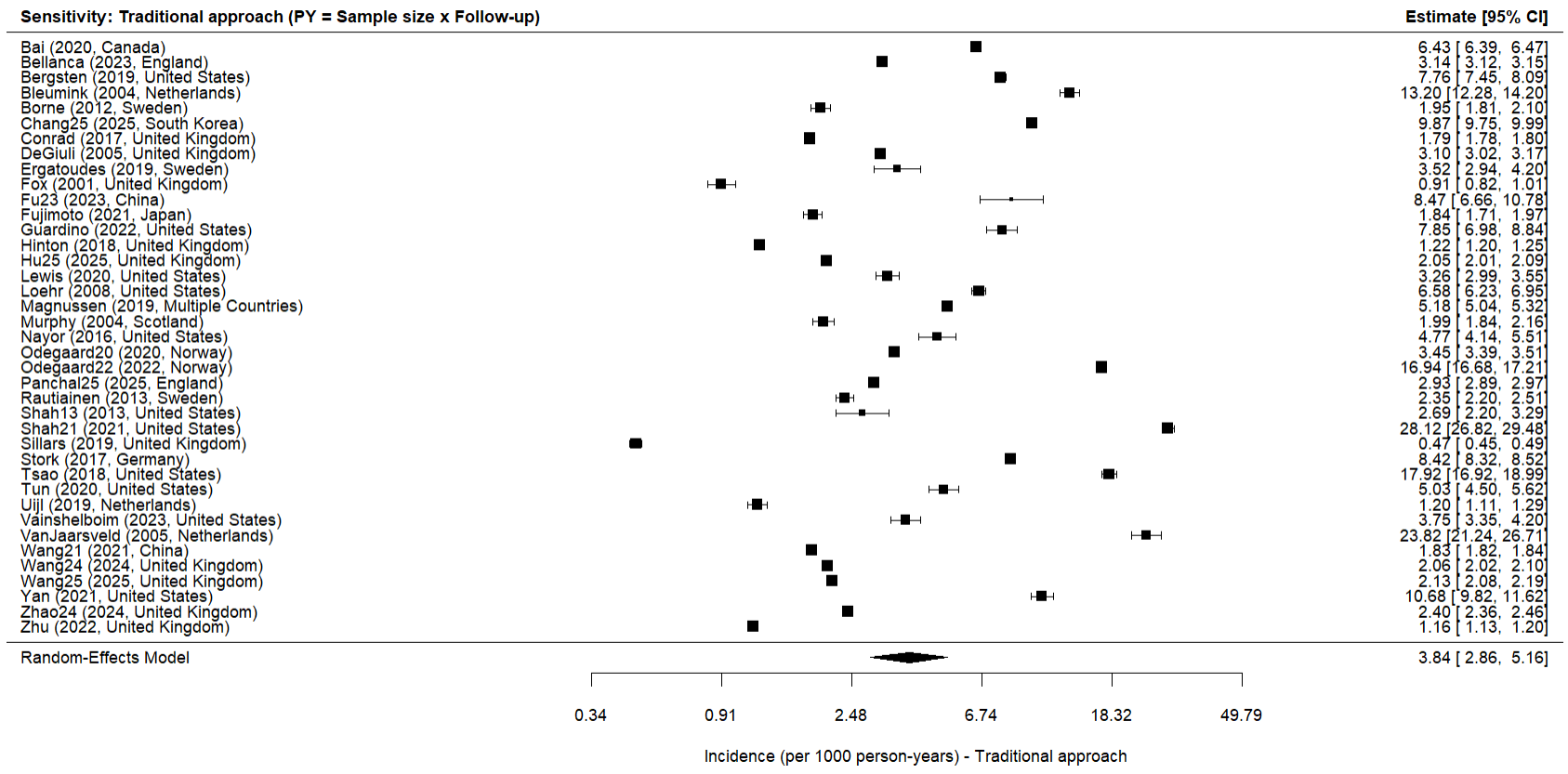


**Supplementary Material 7**. Forest plot of the sensitivity analysis using the traditional approach for person-years calculation (sample size × follow-up time).


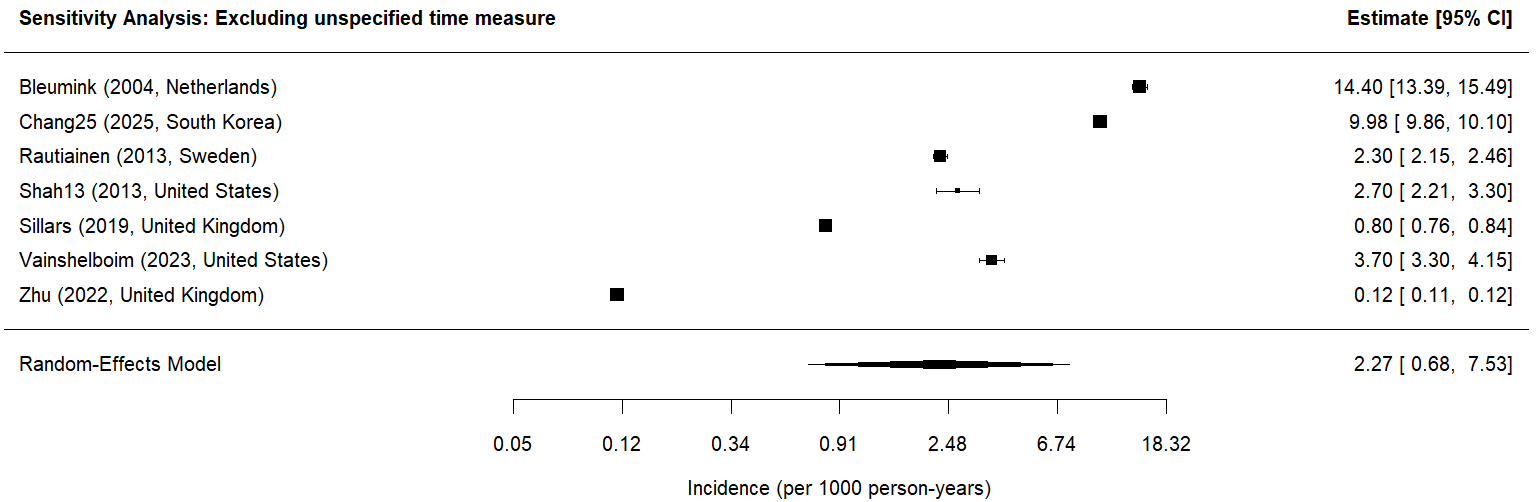


**Supplementary Material 8**. Forest plot of the sensitivity analysis restricted to studies with explicitly defined follow-up time measures (mean or median).

**Supplementary Material 9.** Forest plot of the sensitivity analysis restricted to studies with explicitly defined follow-up time measures (mean or median).

| **Method** | **N** | **Rate per 1000 PY (95%CI)** | **τ²** |
| --- | --- | --- | --- |
| REML (primary) | 42 | 2.72 (1.95–3.81) | 1.2216 |
| DerSimonian-Laird | 42 | 2.72 (1.94–3.83) | 1.2569 |
| REML + Hartung-Knapp | 42 | 2.72 (1.93–3.85) | 1.2216 |
| Paule-Mandel | 42 | 2.72 (1.95–3.81) | 1.2212 |
| Sidik-Jonkman | 42 | 2.72 (1.95–3.81) | 1.2212 |
